# Ejaculatory disorders after prostatic artery embolization: a reassessment of two prospective clinical trials

**DOI:** 10.1007/s00345-019-03036-7

**Published:** 2019-12-07

**Authors:** Gautier Müllhaupt, Lukas Hechelhammer, Pierre-André Diener, Daniel S. Engeler, Sabine Güsewell, Hans-Peter Schmid, Livio Mordasini, Dominik Abt

**Affiliations:** 1 Department of Urology, St. Gallen Cantonal Hospital, Rorschacherstrasse 95, 9007 St. Gallen, Switzerland; 2 Department of Radiology and Nuclear Medicine, St. Gallen Cantonal Hospital, St. Gallen, Switzerland; 3 Clinical Trials Unit, St. Gallen Cantonal Hospital, St. Gallen, Switzerland; 4 Department of Pathology, St. Gallen Cantonal Hospital, St. Gallen, Switzerland

**Keywords:** Anejaculation, Benign prostatic hyperplasia, Diminished ejaculation, Ejaculatory disorders, Prostatic artery embolization, Retrograde ejaculation

## Abstract

**Purpose:**

This study aims to specify and explain the previous findings of unexpectedly high rates of ejaculatory disorders, i.e. 56%, found after prostatic artery embolization (PAE) in a randomized controlled trial comparing safety and efficacy of PAE and transurethral resection of the prostate (TURP).

**Patients and methods:**

Case report forms of the randomized controlled trial were analyzed to specify the grade of postoperative ejaculatory dysfunction 3 months postoperatively.

In addition, study participants with assessable ejaculation were asked to complete the four-item Male Sexual Health Questionnaire-Ejaculation Dysfunction Short Form (MSHQ-EjD) referring to their ejaculatory function at present, as well as before treatment and 3 months after. Potential explanations for ejaculatory disorders after PAE were derived from histological examination of five radical prostatectomy specimens of patients that underwent PAE 6 weeks before radical prostatectomy within a proof-of-concept trial at the study site, St. Gallen Cantonal Hospital. An experienced uropathologist systematically examined the whole-gland embedded tissue with focus on structures that are involved into ejaculation.

**Results:**

While patients after TURP predominantly suffered from anejaculation (52%), diminished ejaculation was found more often after PAE (40%). Significantly higher MSHQ-EjD scores were found 3 months after PAE and at a median follow-up of 31 months.

Histological examination showed marked changes of structures involved into ejaculation (e.g., prostatic glands, seminal vesicles, ejaculatory ducts) after PAE.

**Conclusion:**

Although anejaculation occurs less frequently after PAE (16%) compared to TURP (52%), patients have to be informed about the relevant risk of ejaculatory disorders, especially diminished ejaculation.

## Introduction

Preservation of ejaculatory function is an important issue for many patients undergoing surgery for lower urinary tract symptoms associated with benign prostatic hyperplasia (BPH/LUTS). Reliable preservation of ejaculation is, however, hardly possible with most surgical options, especially if a clear relief of bladder outlet obstruction is anticipated [1].

Prostatic artery embolization was considered to have no influence on ejaculation until recently [2]. In 2018, Ray et al. reported on ejaculatory disorders (referred to as “retrograde ejaculation”) in 24.1% of patients after PAE compared to 47.5% after transurethral resection of the prostate (TURP) in a matched-pair analysis [3]. In our randomized controlled trial comparing PAE and TURP [4], changes in ejaculatory function were assessed according to the CTCAE classification [5] (i.e., grade 1: “diminished”, grade 2: “anejaculation or retrograde ejaculation”). Both grades summarized occurred in 56% (14/25) and 84% (21/25) of patients in whom ejaculation was assessable after PAE and TURP, respectively. These discrepancies compared to previous studies led to confusions and uncertainties in patient counseling.

The present study aims to specify and explain our previous findings by providing more precise data on patients’ ejaculatory function. In addition, we examined post-PAE prostatectomy specimens derived from a proof-of-concept study [6] considering structures involved into ejaculation to assess potential underlying mechanisms of ejaculatory disorders after PAE.

## Patients and methods

Case report forms of the randomized controlled trial were analyzed to specify the grade of postoperative ejaculatory dysfunction (i.e., grade 1: diminished vs. grade 2: anejaculation or retrograde ejaculation) 3 months postoperatively.

In addition, study participants with assessable ejaculation were asked to complete the four-item Male Sexual Health Questionnaire-Ejaculation Dysfunction Short Form (MSHQ-EjD) [7], a validated and abridged version of the 25-item Male Sexual Health Questionnaire (MSHQ) for assessing ejaculatory dysfunction, referring to their ejaculatory function at present, as well as (retrospectively) before treatment and 3 months later. Wilcoxon rank sum tests were used to determine the significance of differences in MSHQ-EjD scores between PAE and TURP.

Potential explanations for ejaculatory disorders after PAE were derived from histological examination of five radical prostatectomy specimens of patients that underwent PAE 6 weeks before radical prostatectomy for localized prostate cancer within a proof-of-concept trial at the study site St. Gallen Cantonal Hospital [6]. An experienced uropathologist (PAD) systematically examined the whole-gland embedded tissue with focus on structures that are involved into ejaculation (i.e., prostatic glands central, peripheral, and adjacent to verumontanum, ejaculatory ducts, seminal vesicles). Tissue changes (i.e., necrosis, fibrosis, hemorrhage) were categorized into none, < 10%, 10–50%, and > 50% and occurrence of particles was categorized to none, few, moderate, and abundant.

The study was approved by the local ethics committee (EKSG 14/004 and BASEC PB_2016-02294).

## Results

**Fig. 1:**
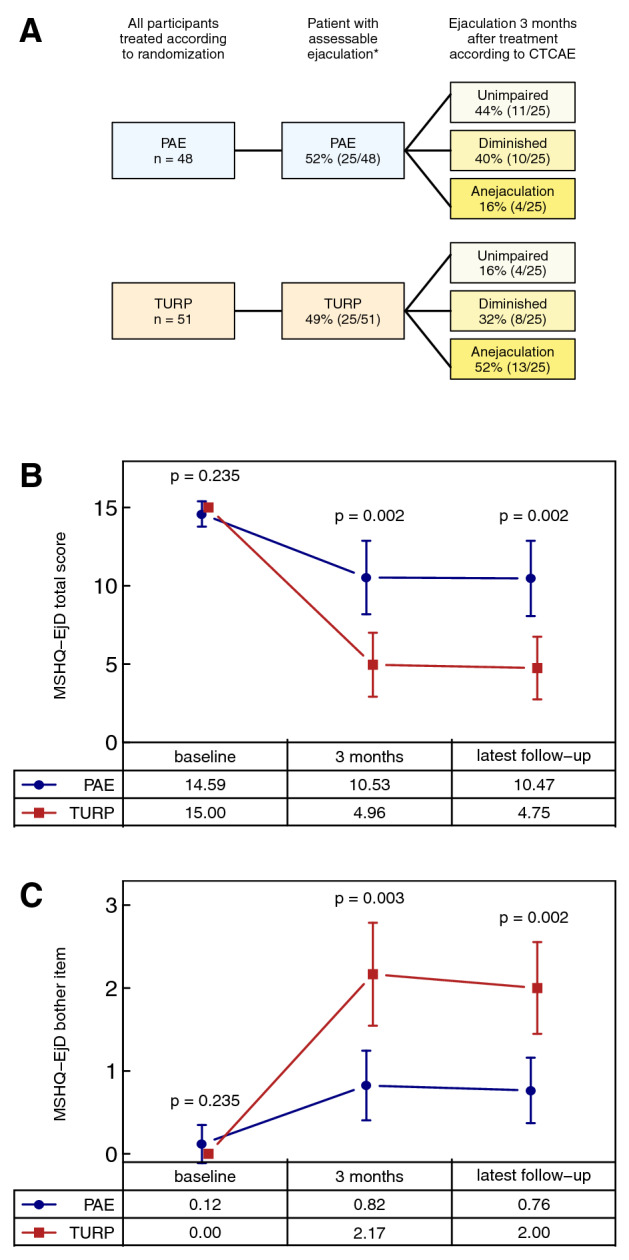
Postoperative ejaculatory disorders assessed according to CTCAE [5] (**a**), Male Sexual Health Questionnaire-Ejaculation Dysfunction Short Form (MSHQ-EjD) ejaculatory function total score (questions 1–3, possible range 0–15) (**b**) and MSHQ-EjD ejaculatory bother item (question 4, possible range 0–5) (**c**) [bars show means and 95% CI, numbers are means, and *p *values from Wilcoxon rank sum tests indicate the significance of differences between PAE and TURP. Asterisk: ejaculation was considered as not assessable in patients with complete erectile dysfunction and in patients reporting to have no sexual stimulation at all; note that MSHQ-EjD was completed retrospectively by the patients for baseline and 3 month follow-up. Latest follow-up was median 31 months (17—58)]

Based on case report forms of the original trial, ejaculatory disorders occurred more frequently and were more pronounced after TURP compared to PAE (Fig. 1a). However, after PAE, 16% and 40% of patients reported on anejaculation or diminished ejaculation, respectively. Baseline characteristics of the patients with assessable ejaculation 12 weeks after intervention are shown in Table 1.

**Table 1:** Baseline characteristics of the patients with assessable ejaculation 12 weeks after intervention.

Characteristic^a^	PAE (*N* = 25)	TURP (*N* = 25)
Age, years	62.9 ± 7.5	63.1 ± 9.0
Body-mass-index, kg m^−2^	25.8 ± 3.5	26.8 ± 3.2
Charlson comorbidity index	3.2 ± 1.2	4.0 ± 2.1
Prostate volume (transabdominal ultrasound), mL	50.6 ± 16.3	52.1 ± 20.1
Prostate volume (magnetic resonance imaging), mL	49.8 ± 31.8	59.7 ± 35.3
Medical treatment of LUTS prior to surgery, no. (%)		
5-Alpha-reductase inhibitors alone	0 (0%)	0 (0%)
Alpha_1_-adrenergic receptor antagonists	12 (48%)	12 (48%)
Combination of the two above	7 (28%)	4 (16%)
Total patients with drug treatment	19 (76%)	16 (64%)
Indwelling urethral catheter at baseline, no. (%)	4 (16%)	3 (12%)

^a^Numeric data are summarized as mean ± standard deviation and categorical data as number and percentage

Forty-one patients were available for the MSHQ-EjD-based survey. Ejaculatory function at baseline was rated as unimpaired by nearly all patients of both groups (Fig. 1b). While, a deterioration of ejaculatory function occurred after PAE and TURP, it was significantly more pronounced after TURP after 3 months and after a median follow-up for 31 months (range 17—58). In accordance, patients were significantly more bothered by ejaculatory disorders after TURP. Thus, the mean rating of bothersomeness after 3 months was between 2 and 3 (“a little bothered” to “moderately bothered”) after TURP compared to less than 1 (“not at all bothered”) after PAE (Fig. 1c).

**Fig. 2:**
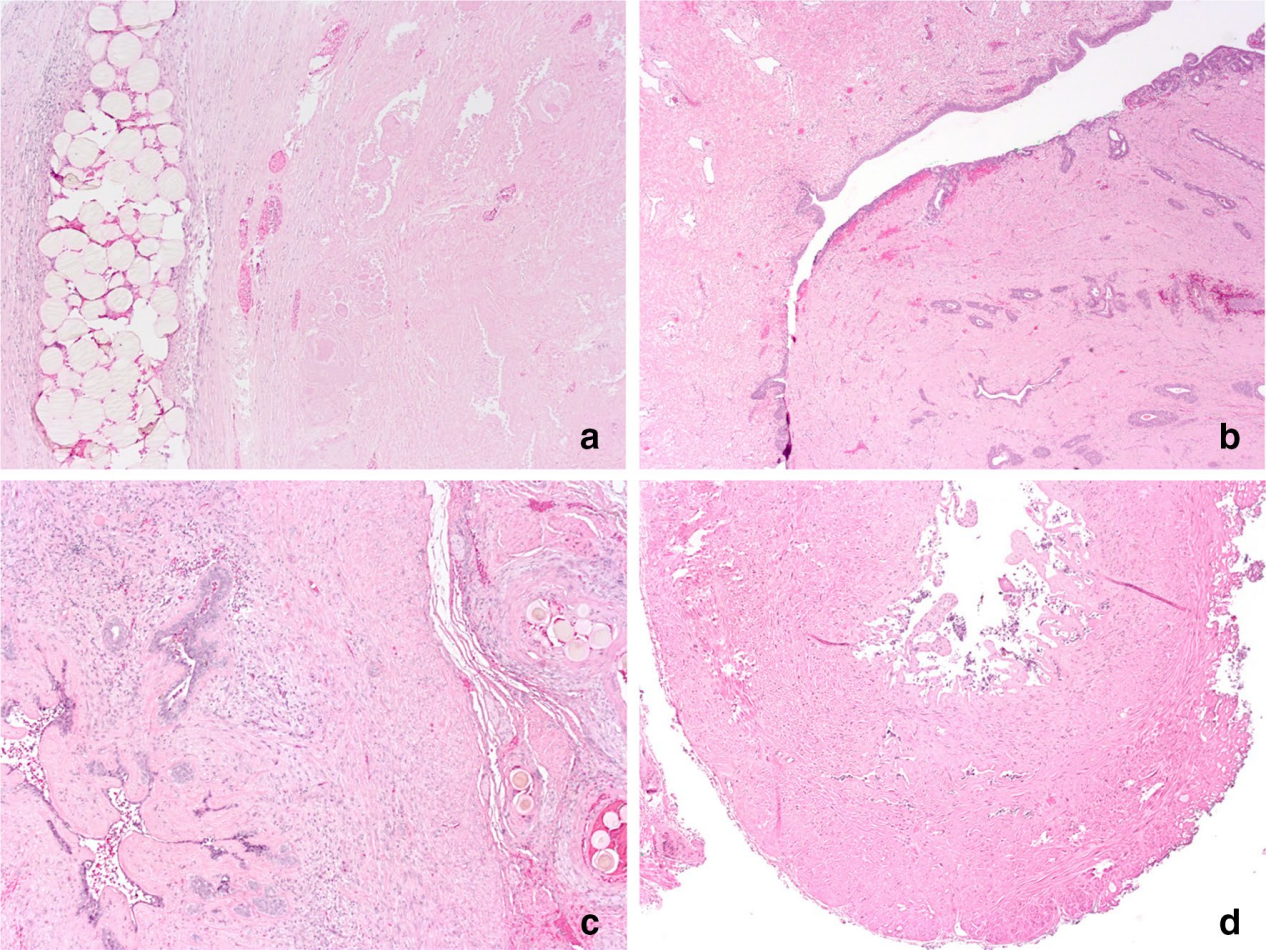
Histological findings in patients undergoing radical prostatectomy 6 weeks after prostatic artery embolization. Selected pictures (HE staining) show extensive necrosis next to embolization particles in the central prostatic gland (**a**, × 50), extensive fibrosis around the verumontanum (**b**, × 25) and mucosal necrosis and atrophy of the seminal vesicles (**c**, × 50) and ejaculatory duct (**d**, × 50)

Histological examination of post-PAE radical prostatectomy specimens revealed extensive fibrosis, necrosis, and hemorrhage. These changes were most distinct at the central gland, but also occurred in all other structures relevant for ejaculatory function in all of the five patients assessed (Table 2, Fig. 2).

**Table 2:** Histological findings of structures involved to ejaculation in five patients undergoing radical prostatectomy 6 weeks after PAE (necrosis/fibrosis/hemorrhage: 0: none, + < 10%, ++ 10–50%, +++ > 50%; occurrence of particles: 0: none, + few, ++ moderate, +++ abundant)

		Patient no.				
Localization	Findings	1	2	3	4	5
Prostate—central gland	Necrosis/fibrosis/hemorrhage	+++	+++	+++	+++ (anterior) ++ (posterior)	+++ (anterior) ++ (posterior)
	Particles	++	+++	++	++	+++ (anterior) ++ (posterior)
Prostate—peripheral gland	Necrosis/fibrosis/hemorrhage	+	++ (anterior) + (posterior)	+++ (anterior) ++ (posterior)	+++ (anterior) ++ (posterior)	++
	Particles	+	+++ (anterior) + (posterior)	+++	++ (anterior) + (posterior)	+++
	Particles in adjacent soft tissue	++	+++ (anterior) ++ (posterior)	+++	+++ (anterior) ++ (posterior)	+ (anterior) +++ (posterior)
Prostate—adjacent to verumontanum	Necrosis/fibrosis/hemorrhage	+	+	++	++	++
	Particles	+	+++	+	++	++
Ejaculatory ducts	Necrosis/fibrosis/hemorrhage	++	++	++	+++	++
	Particles	+	+	0	++	+
	Particles in adjacent soft tissue	++	+	++	0	0
Seminal vesicles	Necrosis/fibrosis/hemorrhage	+++	++	+++	+++	++
	Particles	+	+	+	++	+++
	Particles in adjacent soft tissue	++	+	++	+++	++

## Discussion

This study elucidates the so far sparse and inconsistent data on ejaculatory function after PAE. Although anejaculation occurs less frequently after PAE compared to TURP, patients have to be informed about a substantial risk of ejaculatory disorders, especially diminished ejaculation.

According to the results of our analysis, median bothersomeness of ejaculatory deterioration was rather low in the setting of the original randomized controlled trial. However, preservation of ejaculatory function represents a widely underestimated issue for patients undergoing surgical treatment of BPH/LUTS [8, 9] and, therefore, our results provide important data for patient counseling.

Fibrosis and necrosis of structures involved into ejaculation (Table 1) were identified as possible underlying mechanisms. Considering the fact that these changes were found in all of the structures of each of the patients, absence of ejaculatory disorders in previous trials [2] seems to be caused by underreporting.

Compared to our study, clearly lower rates of ejaculatory disorders have been reported for other minimally invasive treatment options (e.g., Urolift^®^, Rezum^®^) [1]. However, as direct comparisons of PAE with other minimally invasive treatment options are not available and ejaculation preservation rates are highly dependent on the type of assessment performed within a study, further data seem to be mandatory to determine the best treatment for patients aiming at preservation of ejaculatory function.

Strengths of our assessment include the availability of post-PAE radical prostatectomy specimens that allowed for a detailed microscopic assessment and the prospective assessment of ejaculatory changes according to the CTCAE classification within the randomized controlled trial. However, the MSHQ-EjD was not implemented into the study and, therefore, was assessed retrospectively for the baseline and 3 month follow-up visit. As a large proportion of the patients took alpha-blockers, 5-alpha-reductase inhibitors, or a combination of both at the time of the baseline visit (Table 1); baseline MSHQ-EjD-scores seem to be surprisingly high. Thus, ejaculatory function before treatment might has been rated as normal condition or overestimated. Moreover, 100 μm microspheres were used in the proof-of-concept study, from which we obtained tissue samples and histological changes might differ with varying particle sizes.

As ejaculatory function was not in the limelight of our randomized trial, TURP was not performed in an ejaculatory sparing technique, which has to be considered when comparing the results between PAE and TURP. Thus, a significant reduction of ejaculatory disorders has been demonstrated after ejaculation sparing transurethral surgery [1].

## Conclusions

Ejaculatory disorders occur in about 56% of patients undergoing PAE and might result from degenerative changes of anatomical structures involved into ejaculation after PAE. While the majority of these patients develop diminished ejaculation (40%), anejaculation can also occur (16%).
